# The effect of low serum calcium level on the severity and mortality of Covid patients: A systematic review and meta‐analysis

**DOI:** 10.1002/iid3.528

**Published:** 2021-09-17

**Authors:** Effat Alemzadeh, Esmat Alemzadeh, Masood Ziaee, Ali Abedi, Hamid Salehiniya

**Affiliations:** ^1^ Infectious Diseases Research Center Birjand University of Medical Sciences Birjand Iran; ^2^ Department of Medical Biotechnology, Faculty of Medicine Birjand University of Medical Science Birjand Iran; ^3^ Cellular and Molecular Research Center Birjand University of Medical Sciences Birjand Iran; ^4^ Zahedan University of Medical Sciences Zahedan Iran; ^5^ Social Determinants of Health Research Center Birjand University of Medical Sciences Birjand Iran

**Keywords:** calcium, COVID‐19, hypocalcemia, SARS‐CoV‐2, systematic review

## Abstract

**Introduction:**

Imbalances of various electrolytes, including calcium, are associated with the prognosis of Covid disease. This study investigated the relationship between serum calcium and clinical outcomes in patients with COVID‐19.

**Method:**

This study is a systematic review and meta‐analysis by searching PubMed, Scopus, web of sciences until August 2021 using the keywords COVID‐19, severe acute respiratory syndrome coronavirus 2 (SARS‐CoV‐2), COVID, coronavirus disease, SARS‐COV‐infection. 2, SARS‐COV‐2, COVID19, calcium, calcium isotopes, calcium radioisotopes, hypercalcemia, and hypocalcemia were performed. Heterogeneity of studies was investigated using *I* ^2^ index, data were analyzed using meta‐analysis (random effects model) with Comprehensive Meta‐Analysis Software software.

**Results:**

Finally, 25 articles were included in the study. Clinical data from 12 articles showed that 59% (95% confidence interval [CI]: 0.49–0.68) of people with COVID‐19 have hypocalcemia. The results of meta‐analysis showed that hypocalcemia was significantly associated with severity of the disease (*p* = .002), mortality in patients with COVID‐19 (odds ratio [OR] = 6.99, 95% CI: 2.71–17.99), number of hospitalization days (*p* < .001) and admission to the intensive care unit (OR = 5.09, 95% CI: 2.14–12.10). The results also showed that there is a direct relationship between low serum calcium levels with increasing D‐dimer levels (*p* = .02) and decreasing lymphocyte counts (*p* = .007).

**Conclusion:**

Based on the results of meta‐analysis in people with lower calcium, mortality and complications are higher, therefore, serum calcium is a prognostic factor in determining the severity of the disease. Consequently, it is suggested that serum calcium levels should be considered in initial assessments.

## 1 INTRODUCTION

COVID‐19 infection caused by severe acute respiratory syndrome coronavirus 2, named SARS‐CoV‐2, is an infectious disease that spread globally, causing a worldwide pandemic.^1^, ^2^ SARS‐CoV‐2 can be transmitted through contaminated droplets during close contact from person to person (within 1 m)^3^ and primarily has been presented with clinical symptoms including fever, dry cough, headache and myalgia^2^, ^4^. Most patients will experience mild to moderate disease and may recover without requiring critical care, but in high risk individuals with underlying diseases, like chronic respiratory disease, diabetes and cardiovascular disease, infection may lead to a severe syndrome with high lethality.^5^

To date, some studies have reported the relation between biochemical parameters and disease severity. Huang et al., (2019)^2^ showed that COVID‐19 patients admitted to intensive care unit (ICU) had more electrolyte disturbance. The electrolyte unbalance, especially hypocalcemia, is an abnormality in viral infections like MERS‐CoV and SARS‐CoV.^6^, ^7^, ^8^ Calcium is essential ion in cellular processes and metabolic and signaling pathways that play an important role in survival and virulence of viruses.^9^

In COVID‐19, hypocalcemia is highly prominent. Many studies showed that hypocalcemia has a direct relationship with increased likelihood of hospitalization, longer hospitalizations, admission to ICU, ventilation, disease severity, increased D‐dimer and increased mortality in COVID‐19 patients.^10^, ^11^, ^12^, ^13^, ^14^ But published studies in this area have some limitations.^15^ Therefore, a comprehensive study in this regard is necessary. Hence, hypocalcemia in patients with SARS‐CoV‐2 infection with more severe clinical syndrome is not unexpected. So, the degree of hypocalcemia can be a robust metric of disease intensity and is recommended to be considered by physicians in the treatment process. In this study, we analyzed the correlation between admission to ICU and hospital, disease severity, mortality, D‐dimer count, lymphocyte count, and serum calcium levels.

## 2 MATERIALS AND METHODS

### 2.1 Search strategy and information sources

This systematic review and meta‐analysis was based on Preferred Reporting Items for Systematic Reviews and Meta‐Analysis (PRISMA). A comprehensive search was conducted in PubMed, Scopus and Web of Science. Also, to increase the validity of the search, the list of references used in all final articles selected for meta‐analysis was manually searched and evaluated to include other possible sources in the review.

### 2.2 Literature search

For systematic identification, the search was performed based on the following keywords COVID‐19, SARS‐CoV‐2, COVID, Coronavirus Disease 19, SARS CoV 2 Infection, SARS‐CoV‐2, COVID19, Coronavirus Disease, Calcium, Calcium Isotopes, Calcium Radioisotopes, Hypercalcemia, Hypocalcemia in the mentioned databases, using OR, AND. The search was conducted in Aug 17th, 2021; all searched articles were included in the study and retrieved, and EndNote X7 software was used to manage the studies.

### 2.3 Study selection

**Figure 1:**
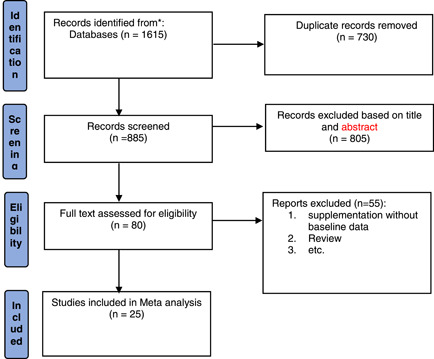
PRISMA flowchart of included studies in the review. PRISMA, Preferred Reporting Items for Systematic Reviews and Meta‐Analysis [Color figure can be viewed at wileyonlinelibrary.com]

After completing the search and entering articles in Endnote software 7, duplicate articles were found by EndNote and removed, then all articles were evaluated by title and abstract, by reading the abstracts, articles related to the effect of calcium and COVID‐19 were entered in this review. PRISMA flow diagram was used for study selection (Figure 1).

### 2.4 Inclusion and exclusion criteria

Inclusion criteria included the following: evaluation of hypocalcemia; mortality, severity, admission to ICU and hospital; D‐dimer and lymphocyte count; articles in English; and all original research articles.

Exclusion criteria were as follows: evaluation of electrolyte abnormalities other than hypocalcemia; hypocalcemia in patients other than the COVID‐19 patients; articles written in languages other than English; case report articles, reviews and letters to the editor; studies that after checking the quality had a low quality.

#### 2.4.1 Data collection tools and methods

Data collection tools was an electronic form consisted of three general sections: (1) general characteristics of the study (first author, type of study design, sample size, age, sex, study place, study quality assessment score and outcome); (2) characteristics of study participants (including median age of participants, male (%), hypocalcemia and normal calcium); (3) methodological information (including number of samples by groups). Excel Microsoft 2016 software was used to facilitate the work.

#### 2.4.2 Measurement of outcome variables

The primary outcome variable of this study (uptake) was the number of hypocalcemia cases among patients with COVID‐19 as well as healthy people. The second outcome variable of this study was identification of correlation between admission to ICU and hospital, disease severity, mortality, D‐dimer count, lymphocyte count, and serum calcium levels.

#### 2.4.3 Risk of bias (quality) assessment

In this systematic review, we used the Newcastle ‐Ottawa Quality Assessment checklist to evaluate the quality of the studies. This tool consists of three separate sections: selection, comparison and outcome. Studies scored based on the overall scores and divided into three categories: Good, Fair, and Poor.^16^

### 2.5 Data analyses

Cochran's Q statistic (significance level of *p* ≤ .1) and *I* ^2^ statistic (significance level of ≥50%) were used to assess statistical heterogeneity. A random effects model was conducted for meta‐analysis of heterogeneous cases (*p* < .1 and *I* ^2^ > 50%), and fixed‐effect model was performed for meta‐analysis of cases without evidence of heterogeneity (*p* > .1 and *I* ^2^ < 50%).

All analyzes were performed using STATA version 13 statistical and comprehensive meta‐analysis software. Studies were analyzed based on odds ratio (OR) and its 95% confidence interval (CI) and standard difference in mean and its 95% CI.

## 3 RESULTS

### 3.1 Selection of studies and study characteristics

After a systematic search, 1615 articles were retrieved in initial search, after removing the duplicates, 885 articles remained, of which 533 articles were irrelevant to hypocalcemia and COVID‐19. The full text of the remaining 80 articles was read and reviewed, and finally a total of 25 articles, including case‐control studies and retrospective cohort studies were included (Figure 1).

The characteristics of the assessed studies are given in Table 1. Clinical data from 12 articles showed that a total of 2891 patients with COVID‐19, of which 1491 had hypocalcaemia, meaning that, decreased serum calcium levels observed in 59% (95% CI: 0.49–0.68) of patients with COVID‐19.

**Table 1:** Characteristic of included studies

First author /study place	Study design	Sample size	Male (%)	Age (median)	Result	Quality^a^
Kashefizadeh (2020) Iran	Retrospective cohort study	Patients: 53	45.3	58.4	Higher mortality rates with Decreased levels calcium	Good
		Expired: 5				
		Survived: 48				
Pal (2021) India	Retrospective case‐control study	Positive SARS‐CoV‐2: 72	–	38	highly prevalent of Hypocalcemia in COVID‐19 patients with non‐severe disease	Good
		Negetive SARS‐CoV‐2: 72				
Raesi (2021) Iran	Case‐control study	Positive SARS‐CoV‐2: 91	60.4	55	Worse clinical outcome and higher mortality rate in patients with lower total serum calcium levels	Good
		Hypocalcemia: 54				
		Normal calcium: 37				
		Negetive SARS‐CoV‐2: 169				
Torres (2021) Spain	Retrospective cohort study	Positive SARS‐CoV‐2: 316	65	65	infection	Good
		Hypocalcemia: 198				
		Normal calcium: 118				
Wu (2020) China	Retrospective study	Positive SARS‐CoV‐2: 125	52.8	55	Hypocalcemia on hospital admission was independent risk factors associated with long‐term hospitalization in patients with COVID‐19.	Good
Cappellini (2020) Italy	Retrospective study	Positive SARS‐CoV‐2: 420	65.9	66	Total serum calcium decrease with increasing age	Good
		Negetive SARS‐CoV‐2: 165				
Yang (2020) China	Retrospective observational study	Positive SARS‐CoV‐2: 226	60.6	40	Low calcium could indicate the severity of COVID‐19 patients	Good
		Suspected cases: 122				
		Confirmed cases: 104				
		Moderate: 68				
		Critical: 36				
Zhou (2020) China	Retrospective study	Positive SARS‐CoV‐2: 127	–	–	Low calcium is a common abnormal parameter in COVID‐19 patients regardless of the severity	Good
		Moderate: 82				
		Severe: 45				
Ucciferri (2021) Italy	Retrospective observational study	Positive SARS‐CoV‐2: 280	53.5	72	The evaluation of laboratory exams and radiological characteristics are still a fundamental tool to identify COVID‐19 cases	Good
		Negetive SARS‐CoV‐2: 286				
Liu (2020) China	Retrospective study	Positive SARS‐CoV‐2: 107	49	68	Hypocalcemia commonly occurred in severe COVID‐19 patients and it was associated with poor outcome	Good
		Hypocalcemia: 67				
		Normal calcium: 40				
Zhao (2021) China	Retrospective cohort study	Positive SARS‐CoV‐2: 172	47.7	65	Calcium level can be used to a prognostic prediction of severity for patients with moderate COVID‐19 in the early stage of the disease	Good
		Mild: 112				
		Severe: 60				
Osman (2021) Oman	Observational cohort study	Positive SARS‐CoV‐2: 445	62.0	50.8	Hypocalcemia is a significant and reliable marker of disease severity	Good
		Hypocalcemia: 306				
		Normal calcium: 139				
Sun (2020) China	Retrospective study	Positive SARS‐CoV‐2: 241	46.5	65	Serum calcium was associated with the clinical severity and prognosis of patients with COVID‐19	Good
		Hypocalcemia: 180				
		Normal calcium: 61				
Bennouar (2020) Algeria	Prospective study	Positive SARS‐CoV‐2: 120	69.2	62.3	The high frequency of hypocalcemia in severe COVID‐19 patients.	Good
		Hypocalcemia: 43				
		Normal calcium: 77				
Lu (2020) China	Retrospective multicenter study	Positive SARS‐CoV‐2: 304	59.9	44	hypocalcemia is associated with the clinical severity of patients with COVID‐19	Good
		Moderate: 112				
		Severe: 60				
Tezcan (2020) Turkey	Retrospective observational study	Positive SARS‐CoV‐2: 408	46.1	54.3	Baseline electrolyte abnormalities are a sign of unfavourable prognosis in COVID‐19 and would be beneficial to assessing the risk for severe COVID‐19.	Fair
		Hypocalcemia: 181				
		Normal calcium: 227				
Filippo (2020) Italy	Retrospective cohort study	Positive SARS‐CoV‐2: 531	67.8	59	hypocalcemia is highly incident in COVID‐19 patients and predicts the need for hospitalization	Good
		Hypocalcemia: 462				
		Normal calcium: 69				
Abdolahi Shahvali (2021) Iran	Case‐control study	Positive SARS‐CoV‐2: 93	44.1	51	Calcium levels in COVID‐19 patients are lower than control group.	Good
		Hypocalcemia: 39				
		Normal calcium: 54				
		Negetive SARS‐CoV‐2: 186				
Cap (2021) Turkey	Retrospective observational study	Positive SARS‐CoV‐2: 132	53	50	Significant relationship was observed between iCa levels and the QTc interval	Good
		Hypocalcemia: 76				
		Normal calcium: 56				
Dubey (2021) North India	Retrospective pilot study	Positive SARS‐CoV‐2: 200	73.5	45.79	Serum biochemistry parameters could be used as a screening tool to identify patients requiring intensive care	Good
Filippo (2021) Italy	Retrospective cohort study	Positive SARS‐CoV‐2: 20	50	73	hypocalcemia may be a distinctive biochemical feature of COVID‐19 potentially impacting on disease clinical severity	Good
		Hypocalcemia: 16				
		Negetive SARS‐CoV‐2: 20				
		Hypocalcemia: 8				
Jahangirimehr (2021) Iran	Cross‐sectional study	Positive SARS‐CoV‐2: 93	44	51	The severity of COVID‐19 in patients was significantly different according to serum calcium levels (*p* = .005) The lower the calcium level, the more severe the disease	Good
		Low: 26				
		Moderate: 30				
		Severe: 37				
Mahmood (2021) Pakistan	Retrospective study	Negetive SARS‐CoV‐2: 140	52.5	60.91	Close monitoring of blood calcium levels can predict the severity of the disease more effectively	Fair
		Positive SARS‐CoV‐2: 40				
		Hypocalcemia: 67				
		Normal calcium: 113				
Qi (2021) China	Not Mentioned	Positive SARS‐CoV‐2: 107	50.46	48.86	Decreased Ca2^+^ and coagulation dysfunction in COVID‐19 patients were significantly correlated with each other and with inflammatory factors.	Good
		Mild: 30				
		Severe: 30				
Zheng (2021) China	Retrospective	Positive SARS‐CoV‐2: 180	62.7	64	More nonsurvivors than survivors presented with a low serum calcium concentration. Therefore, hypocalcaemia might be a predictor of severe disease.	Good

^a^ Newcastle ‐Ottawa Quality Assessment Form (1).

Among the meta‐analysis evaluated studies, seven studies have examined serum calcium levels in healthy individuals and patients with COVID‐19.^10^, ^13^, ^17^, ^18^, ^19^, ^20^, ^21^ Also, the relationship between mortality and hypocalcemia in eight articles evaluated,^9^, ^12^, ^13^, ^22^, ^23^, ^24^, ^25^, ^26^ in addition, the relationship between severity of the disease and hypocalcaemia in seven articles evaluated,^13^, ^22^, ^27^, ^28^, ^29^, ^30^, ^31^ and the relationship between hypocalcaemia and the length of hospital stay and admission in the ICU is reported in four and five articles, respectively.^9^, ^12^, ^13^, ^19^, ^22^, ^32^ Also, four articles reported hypocalcaemia association with D‐dimer level and six articles reported that hypocalcaemia is related to lymphocyte count.^9^, ^12^, ^23^, ^33^, ^34^

The mean serum calcium level in the group of patients with COVID‐19 was significantly lower than healthy individuals (difference between the mean of the patient group compared to the healthy group was 0.44 and *p* < .001).

**Figure 2:**
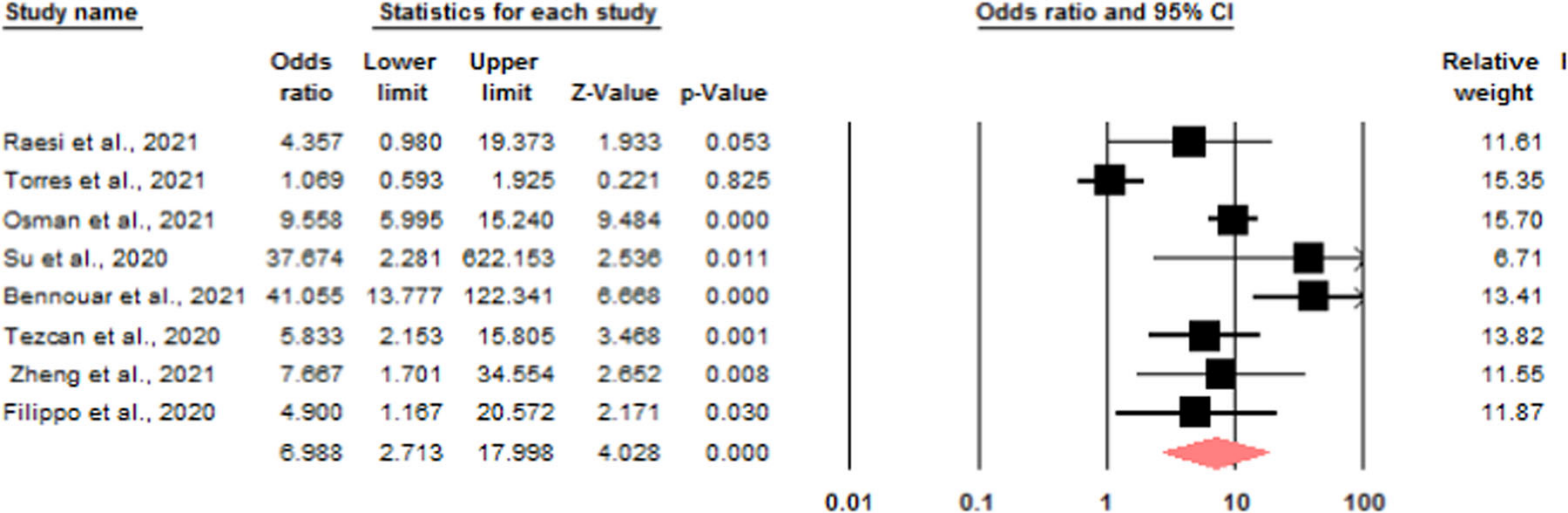
Odds ratio for low serum calcium levels and mortality [Color figure can be viewed at wileyonlinelibrary.com]

Mortality: eight studies were eligible to investigate the relationship between serum calcium levels and death of patients due to COVID‐19. Due to the heterogeneity (*I* ^2^ = 86.1) a random model was used to investigate the relationship, the chance of death ratio in the group of patients with low serum calcium levels compared to the group of patients with normal calcium levels is 6.98 with a CI range of 2.71–17.99 (Figure 2).

Severity of the disease: In terms of mean calcium levels, there is a significant difference between the two groups with severe and mild symptoms (*p* = .002), so that in the group with mild symptoms, serum calcium levels are higher than that in patients with severe symptom.

Hospitalization: The standard difference in mean of hospitalization in the group of patients with lower serum calcium levels is longer than the group with normal serum calcium (*p* < .001).

**Figure 3:**
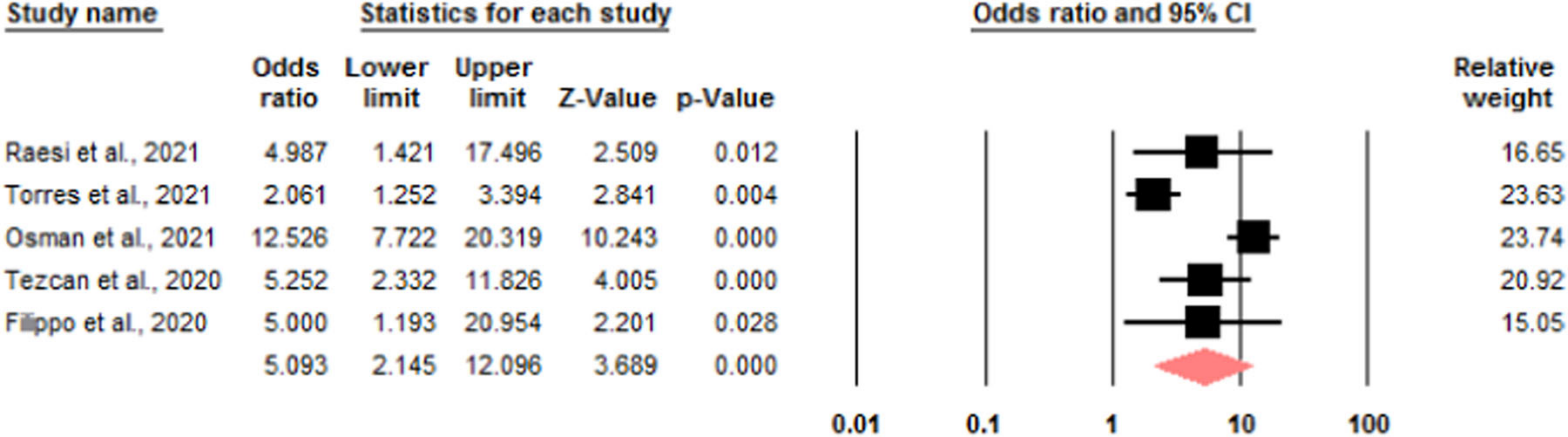
Odds ratio for low serum calcium levels and intensive care unit (ICU) admission [Color figure can be viewed at wileyonlinelibrary.com]

ICU admission: five studies were eligible to investigate the relationship between serum calcium levels and ICU admission of patients with COVID‐19. Due to the heterogeneity (*I* ^2^ = 0.84), a random model was used to investigate the relationship. The odds ratio of admission to ICU in the group of hypocalcemia patients compared to the group of patients with normal serum calcium is 5.09 with a CI of 2.14–12.09 (Figure 3).

D‐diameter level: due to the presence of heterogeneity (*I* ^2^ = 82.4), a random model was used. The standard difference in means between the two groups is different (*p* = .02), although the level of D‐dimer is higher in the group of hypocalcemia patients, this is not significant.

Lymphocyte count: based on the results, the mean difference between the two groups of patients with low and normal serum calcium is 0.273 which shows that there is a significant difference in the number of lymphocytes (*p* = .007). In other words, in the group of hypocalcemia patients, the number of lymphocytes has significantly decreased.

## 4 DISCUSSION

In many viral diseases, serum calcium levels fall to less than 2.2 mmol/L, which is called hypocalcemia. Hypocalcemia is a common abnormality in viral infections such as SARS, Ebola, pneumonia, and the recent SARS‐COV‐2 pandemic.^2^, ^35^

Numerous studies have reported severe hypocalcemia in more than 60% of patients with COVID‐19 hospitalizations.^1^, ^3^, ^4^, ^5^, ^6^, ^7^, ^36^ Therefore, it has been hypothesized that there is a relationship between hypocalcemia and the severity of COVID‐19 disease. The aim of this study was to evaluate the serum calcium level in patients with COVID‐19 and healthy individuals and its relationship with severity of the disease and mortality in patients with COVID‐19. According to the results, calcium levels in patients with COVID‐19 are significantly lower than in healthy individuals. The cause of hypocalcemia in COVID‐19 patients with a critical condition is not known exactly, but several mechanisms have been suggested for this: (1) Old age and chronic malnutrition that led to vitamin D deficiency can lead to hypocalcemia. (2) Calcium is mainly bound to plasma albumin. Decreased serum albumin levels can cause hypocalcemia. (3) Proinflammatory cytokines in patients with COVID‐19 inhibit parathyroid hormone (PTH) secretion and cause calcium imbalance. (4) Elevated levels of unsaturated fats have been reported in patients with COVID‐19, since unsaturated fatty acids can bind to calcium and cause hypocalcemia.^8^, ^9^, ^10^

### 4.1 The relationship between hypocalcemia and severity of disease and increased mortality in patients with COVID‐19

The results showed that the disease is less severe in patients with lower serum calcium levels and mortality is significantly higher among these patients. Due to the fact that calcium homeostasis is completely regulated by hormonal processes, especially PTH, the mechanism by which the virus uses calcium for reproduction and survival does not fully explain the low serum calcium levels observed in SARS‐CoV‐2 infection. Therefore, there may be a relationship between hypocalcemia and inflammation. According to some studies, cytokines can impair the expression of calcium receptor and thus cause an imbalance in serum calcium levels.^37^ Numerous studies have shown that inflammatory markers, including cytokines, are higher in the serum of COVID‐19 patients than in healthy individuals.^38^, ^39^ Proinflammatory cytokines such as interleukin‐1 and interleukin‐6 are produced in critical disease conditions. The association between hypocalcemia and more severe infection and consequent higher mortality can be explained by the interaction between serum calcium levels and the immune system.^40^, ^41^. The findings of this study also confirmed that serum calcium levels are directly related to severity of disease and mortality in patients with COVID‐19. These findings in the present study are consistent with the findings of Martha (2021).^36^

### 4.2 Relationship between hypocalcemia and lymphocyte count and D‐dimer level in patients with COVID‐19

The results of meta‐analysis showed that there was a significant difference between the two groups of patients with low and normal calcium levels in terms of lymphocyte count and D‐dimer level. Patients with low serum calcium have fewer lymphocytes, higher D‐dimer levels, and therefore, more severe disease. Studies have shown that the SARS‐COV‐2 virus may also kill cytotoxic T lymphocytes by mechanisms similar to acute respiratory syndrome (SARS), such as direct bone marrow suppression or destruction by cytokines. T lymphocytes are essential to control viral infections.^12^, ^13^, ^22^ The results of D‐dimer analysis in the present study showed that there is a direct relationship between D‐dimer‐dimer level and hypocalcemia. The lower the serum calcium level, the significantly higher the D‐dimer level and consequently the severity of the disease. Paliogiannis et al. (2020)^42^ reported in a meta‐analysis study that D‐dimer levels were significantly higher in patients with COVID‐19 and were directly related to severity of the disease.^42^ Therefore, two factors, lymphocyte count and D‐dimer level, are directly related to severity of the disease and serum calcium levels. Patients with hypocalcemia have lower lymphocytes and higher D‐dimer levels, have more inflammatory response. Therefore, calcium levels can be a predictor of severity of the disease.

### 4.3 Relationship between hypocalcemia and the number of hospitalization days and admission to the ICU

The results of the present meta‐analysis revealed that hypocalcemia in patients with COVID‐ 19 was significantly associated with an increase in the number of hospitalization days and the probability of admission to the ICU. Numerous studies have examined the risk factors for hospitalization of patients with COVID‐19, including high CRP, old age, low lymphocyte count, elevated cytokine levels, and hypoalbuminemia. Increased levels of cytokines and hypoalbuminemia are factors that can cause hypocalcemia and increase the length of hospital stay. Regardless of the mechanism responsible for hypocalcemia, low calcium levels appear to be recurrent in hospitalized patients with SARS‐CoV‐2 infection and can have important clinical consequences, including cardiovascular, neurological, and musculo‐skeletal and mental side effects. Therefore, attention to this biochemical marker is of special importance.^24^, ^43^

## 5 CONCLUSION

The results of meta‐analysis showed that patients with lower calcium levels, severity of disease was higher and hospitalization and mortality were higher. Due to high mortality and lack of definitive treatment in critical patients, early detection of prognostic parameters of patients is (Wang et al. 2020^5^) necessary for intervention, so the role of serum calcium levels as a prognostic biochemical marker in determining the severity of the disease is important. Since it is easy to measure serum calcium in emergencies and helps physicians identify the severity of the disease, it is recommended that serum calcium levels should be considered in initial assessments.

## CONFLICT OF INTERESTS

The authors declare that there are no conflict of interests.

## AUTHOR CONTRIBUTIONS

**Hamid Salehiniya, Effat Alemzadeh, and Masood Ziaee**: conceptualized and designed the study. **Esmat Alemzadeh, Hamid Salehiniya, and Ali Abedi**: participated in data collection and data collection. **Hamid Salehiniya and Esmat Alemzadeh**: analyzed the data. **Effat Alemzadeh, Esmat Alemzadeh, Ali Abedi, and Hamid Salehiniya**: coordinated the writing of the manuscript. All of the authors read the initial manuscript, commented on all parts of the text, and approved the final version.

## Data Availability

The datasets generated and analyzed for the study are available from the corresponding author with permission from Birjand University of Medical Science, Birjand, Iran.
